# Protein Intake Falls below 0.6 g·kg^-1^·d^-1^ in Healthy, Older Patients Admitted for Elective Hip or Knee Arthroplasty

**DOI:** 10.1007/s12603-019-1157-2

**Published:** 2019-01-23

**Authors:** M. E. G. Weijzen, I. W. K. Kouw, A. A. J. Verschuren, R. Muyters, J. A. Geurts, P. J. Emans, P. Geerlings, L. B. Verdijk, L. J. C. van Loon

**Affiliations:** 1 Department of Human Biology, School of Nutrition and Translational Research in Metabolism (NUTRIM), Maastricht University Medical Centre+, PO Box 616, 6200 MD Maastricht, the Netherlands; 2 Daily Fresh Food, Geleen, the Netherlands; 3 Department of Orthopedic Surgery, Care and Public Health Research Institute (CAPHRI), Maastricht University Medical Centre+, Maastricht, the Netherlands; 4 Department of Dietetics, Maastricht University Medical Centre+, Maastricht, the Netherlands

**Keywords:** protein, consumption, hospitalization, hospital meals

## Abstract

**Objective:**

Hospitalization is generally accompanied by changes in food intake. Patients typically receive hospital meals upon personal preference within the framework of the food administration services of the hospital. In the present study, we assessed food provision and actual food and snack consumption in older patients admitted for elective hip or knee arthroplasty.

**Design:**

A prospective observational study.

**Setting:**

Orthopedic nursing ward of the Maastricht University Medical Centre+.

**Participants:**

In the present study, n=101 patients (age: 67±10 y; hospital stay: 6.1±1.8 d) were monitored during hospitalization following elective hip or knee arthroplasty.

**Measurements:**

Energy and protein provided by self-selected hospital meals and snacks, and actual energy and protein (amount, distribution, and source) consumed by patients was weighed and recorded throughout 1–6 days.

**Results:**

Self-selected meals provided 6.5±1.5 MJ·d^-1^, with 16, 48, and 34 En% provided as protein, carbohydrate, and fat, respectively. Self-selected hospital meals provided 0.75±0.16 and 0.79±0.21 g·kg^-1^·d^-1^ protein in males and females, respectively. Actual protein consumption averaged merely 0.59±0.18 and 0.50±0.21 g·kg^-1^·d^-1^, respectively. Protein consumption at breakfast, lunch, and dinner averaged 16±8, 18±9, and 20±6 g per meal, respectively.

**Conclusions:**

Though self-selected hospital meals provide patients with ∼0.8 g·kg^-1^·d^-1^ protein during short-term hospitalization, actual protein consumption falls well below 0.6 g·kg^-1^·d^-1^ with a large proportion (∼32%) of the provided food being discarded. Alternative strategies are required to ensure maintenance of habitual protein intake in older patients admitted for elective orthopedic surgery.

**Electronic Supplementary Material:**

Supplementary material is available for this article at 10.1007/s12603-019-1157-2 and is accessible for authorized users.

## Introduction

Hospitalization in older adults is accompanied by substantial changes in food intake. At present, the hospitalization duration in older patients is 5 days or longer (1, 2). During such a short period of hospitalization, food intake is generally reduced due to periods of fasting, adverse effects of medication, strict timing of food provision, reduced appetite, and/or pain and discomfort (3-5). Such a reduced food intake throughout the hospitalization period often results in a negative energy and/or protein balance. Low levels of energy and protein intake, also referred to as protein-energy malnutrition, result in accelerated loss of lean body mass, muscle strength, and impairments in functional capacity (6-8). Moreover, malnutrition during hospitalization has been shown to increase the length of hospital stay, the risk for infections, the incidence of hospital readmissions, and mortality rates (9-12).

The negative health consequences of malnutrition are, at least partly, attributed to the concomitant loss of skeletal muscle mass and strength. Several studies from our group, as well as others, have shown significant declines in muscle mass and strength during short periods of immobilization (13-18) and hospitalization (19, 20). It has been wellestablished that the loss of muscle tissue is accelerated when energy balance remains negative (21). Apart from the negative effects of physical inactivity, the loss of muscle mass observed during hospitalization may be particularly attributed to an insufficient protein intake as a direct consequence of the lower energy intake. The current recommended dietary intake for protein has been set at 0.8 g•kg^-1^•d^-1^ for healthy adults of all ages. In the general population, older communitydwelling individuals consume well above (~1.0 g•kg^-1^•d^-1^) these recommended protein intake levels (6, 22-24). Recently updated guidelines suggest a protein intake of 1.2-1.5 g•kg^-1^•d^-1^ for older individuals suffering from acute or chronic diseases in order to maintain muscle mass during hospitalization (25, 26). Minimal requirements during hospitalization should be aimed at achieving energy balance and maintaining habitual protein intake. Currently, patients typically receive hospital meals upon personal request within the framework of the food administration services of the hospital. However, it is unknown whether these self-selected hospital meals provide sufficient energy and/or protein.

Whereas several studies have assessed energy and/or protein provision in patients during hospitalization (3, 9, 27-33), only few studies have measured the actual amount of energy and protein consumed by patients (4, 34-38). In addition, snack consumption between meals is generally not reported. Recent studies show that less than 30% of the older hospitalized patients reach a protein intake of 1.2 g•kg^-1^•d^-1^ (36, 39). Moreover, protein intake has been shown to be well below these increased recommendation levels for at least one day in older patients during hospitalization (36, 38). There is no data available on actual energy and protein intake during the entire hospitalization period in older patients. In addition, a clear quantification on the protein consumption pattern in hospitalized patients such as the protein amount per meal, protein distribution, and protein sources has not been reported. We hypothesized that older patients during several days of hospitalization consume well below the recommended protein intake level of 1.2-1.5 g•kg^-1^•d^-1^.

In the present study, we assessed food provision as well as actual food and snack consumption in older patients (n=101) during short-term hospitalization following elective total hip or knee arthroplasty. We quantified the amount, distribution, and source of protein in self-selected hospital meals consumed at breakfast, lunch, dinner, and snacks during several days of hos pitalization.

## Materials and methods

### Study design

This observational study assessed the nutritional content of self-selected hospital meals, and measured actual food and snack consumption in all patients undergoing elective hip or knee arthroplasty between April 2016 and August 2016. Patients were screened for malnutrition using the Malnutrition Universal Screening Tool (MUST) (40) upon arrival on the nursing ward as part of standard admission procedures (all included patients had a MUST=0 score upon hospital admission). Information concerning the project was given orally and patients gave consent to collect their food trays after meal consumption. Age, BMI, type of anesthesia used during surgery, and length of stay (LOS) were recorded. There was no extra burden on the patient during hospitalization. There were no exclusion criteria. The study was registered as NTR5942 (www.trialregister.nl). Observational food intake data and retrospective, blinded patient data were collected under the Agreement on Medical Treatment Act and the Personal Data Protection Act, according to Medical Ethical standards.

### Provision of hospital meals

Hospital meals were provided at three strict timeslots every day; at breakfast, lunch, and dinner. In between the main meals, patients were provided with hot and/or cold drinks 3 times a day. There was mealtime assistance during the provision of all meals. Patients selected their meals upon request the day before and could indicate different portion sizes of 0.5, 1, or 2 portion(s). During the first day after surgery, when patients were not able to select their meals due to the time spent at the surgery room, they received standard hospital meals.

### Consumption of hospital meals

On the serving tray, patients received a description of their ordered menu, which was collected for the study. When patients were finished eating, the serving tray was taken and all leftovers were weighed using a scale (Soehnle, Germany) and reported. To assess snack consumption, patients were asked to fill in a daily snack list during hospitalization that was collected daily. If patients were not able to complete the snack list, the researcher recalled their snack consumption in between meals and reported this. During the entire assessment period the researcher was present at the ward.

### Nutritional content of hospital meals

Total energy (MJ), protein (g and En%), carbohydrate (g and En%), and fat (g and En%) were calculated for all provided and consumed food based upon product specifications provided by the food suppliers and the Dutch Food Consumption Database 2016 (NEVO; RIVM, Bilthoven, the Netherlands) (41). The contribution of animal- and plant-based sources to dietary protein intake was determined and expressed as a percentage of total protein intake for all meals. Food intake was recorded from the day of hospital admission (day 0) until the day of hospital discharge (day 2-6). Data on food intake on the day of hospital admission and discharge was not included, since these days did not include all main meals. The reported intake was calculated in n=101 patients from the day of surgery (day 1) until day 2, in n=98 until day 3, in n=52 until day 4, and in n=17 until day 5.

To estimate patients’ nutritional needs, energy requirements were calculated based upon resting energy expenditure using the Harris and Benedict equation (42). A Physical Activity Level (PAL) of 1.3 for patients “not restricted to bed” and an Injury Factor (IF) of 1.2 for “minor operation” was used, resulting in a correction factor of 1.56 to estimate (minimal) energy requirements (43). During hospital stay patients were encouraged to mobilize as soon as possible and received physiotherapy training for 30 min daily, from day 2 onwards.

### Statistical analysis

**Figure 1:**
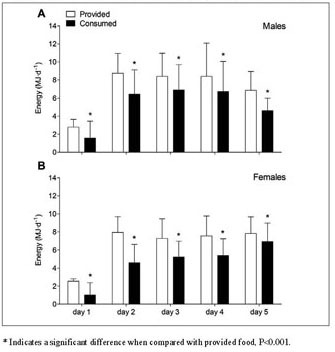
Mean (±SD) energy provision and consumption (MJ•d^-1^) during short-term hospitalization in older, hospitalized males (A) and females (B). Food intake was calculated in n=101 (M/F 37/64) patients until day 2, in n= 98 (M/F 36/62) until day 3, in n=52 (M/F 17/35) until day 4, and in n=17 (M/F 6/11) until day 5

**Figure 2:**
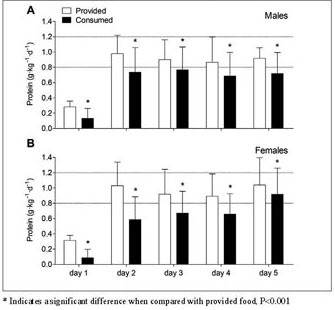
Mean (±SD) protein provision and consumption (g•kg^-1^•d^-1^) during short-term hospitalization in older, hospitalized males (A) and females (B). Food intake was calculated in n=101 (M/F 37/64) patients until day 2, in n= 98 (M/F 36/62) until day 3, in n=52 (M/F 17/35) until day 4, and in n=17 (M/F 6/11) until day 5. The dotted lines represent the recommended dietary intake of 0.8 g•kg^-1^•d^-1^ and the recommended protein intake of 1.2 g•kg^-1^•d^-1^ suggested for older, hospitalized individuals

**Figure 3:**
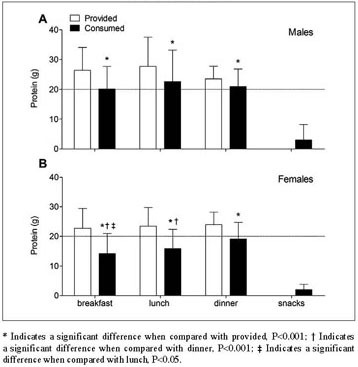
Mean (±SD) dietary protein provision and consumption (g) across main meals in older, hospitalized males (A; n=37) and females (B; n=64)

All data was checked for normality and was normal distributed, except for energy and protein consumption on day of surgery (day 1). Data are expressed as mean±SD (for consistency; both normal and non-normal distributed data). Differences between provided and consumed food intake were analyzed using a paired Students t-test. Energy and protein intake during hospitalization and between main meals were analyzed using repeated measures ANOVA with time as withingroup factor (either days or meals) and gender as betweengroup factor. Analyses for energy and protein intake were performed for patients hospitalized until day 3 (Figure 1 and Figure 2) and analyses for protein intake distribution were performed excluding snack consumption (Figure 3). In case of a significant interaction between time and gender, separate analyses were performed to determine time-effects for males and females (one-factor repeated measures ANOVA) with a Bonferroni post-hoc test to locate these differences and between-group effects for each time-point (Students t-test). Statistical significance was set at P<0.05. All calculations were performed using the statistical software program SPSS (version 24.0, IBM Corp., Armonk, USA).

## Results

### Patients’ characteristics

In total, 101 older patients were monitored (males/ females: 37/64; age: 67±10 y; hospital stay: 6.1±1.8 d) during hospitalization following elective hip or knee arthroplasty. Patients’ characteristics are presented in Table 1. Estimated resting metabolic rate averaged 7.15±0.74 MJ•d^-1^ in males and 5.81±0.87 MJ•d^-1^ in females. The estimated (minimal) energy requirements were calculated using a correction factor of 1.56 and averaged 11.15±1.16 MJ•d^-1^ in males and 9.06±1.35 MJ•d^-1^ in females.

**Table 1:** Patients’ characteristics

	**All patients**	**Males**	**Females**
	**(n=101)**	**(n=37)**	**(n=64)**
Age (y)	67±10	66±8	68±10
Body weight (kg)	81.1±16.9	86.3±11.0	78.1±18.9*
Height (m)	1.69±0.09	1.75±0.07	1.65±0.08*
BMI ((kg•m-2)	28.4±5.0	28.3±3.6	28.5±5.7
Length of stay (d)	6.1±1.8	6.1±1.8	6.1±1.9
Resting Metabolic Rate (MJ•d-1)	6.30±1.04	7.15±0.74	5.81±0.87*
General Anesthesia (n)	68 (67%)	25 (68%)	43 (67%)
Spinal Anesthesia (n)	33 (33%)	12 (32%)	21 (33%)
THA (n and %)	57 (56%)	18 (49%)	39 (61%)
TKA (n and %)	44 (44%)	19 (51%)	25 (39%)

Values are mean±SD. BMI: body mass index, THA: total hip arthroplasty, TKA: total knee arthroplasty. Resting metabolic rate was calculated based upon gender, body weight, height and age, using the adjusted Harris and Benedict equation. Data were analyzed by a Students t-test. * Indicates a significant difference between males and females, P<0.05

### Energy provision and consumption

Energy provision and consumption (MJ•d^-1^) from selfselected hospital meals during hospitalization are presented in Figure 1. Hospital meals provided 6.90±1.78 MJ•d^-1^ in males (Figure 1A) and 6.25±1.34 MJ•d^-1^ in females (Figure 1B) during the entire hospitalization period, while energy consumption averaged 5.29±1.77 and 3.96±1.39 MJ•d^-1^, respectively. On average, 32±17% of the provided food (i.e. energy content) was not consumed (P<0.001). For energy intake, a significant time x gender interaction was observed (P<0.001). For both males and females, energy intake increased from day 1 (1.58±1.87 and 1.02 ±1.38 MJ•d^-1^) to day 2 (6.45±2.66 and 4.60±2.00 MJ•d^-1^; P<0.001), with a further increase observed to day 3 (6.92±2.77 and 5.24±1.73 MJ•d^-1^) although this only reached significance for females (P=0.001). Energy consumption was lower in females when compared with males on day 2 and 3 of hospitalization (both P<0.001) and tended to be lower in females on day 1 (P=0.084). Total macronutrient consumption (g and En%) in males and females are presented in Table 2.

**Table 2:** Macronutrient intake in older, hospitalized males (n=37) and females (n=64)

	**Energy (MJ)**		**Carbohydrate (g and (En%))**		**Protein (g and (En%))**		**Fat (g and (En%))**	
	Males	Females	Males	Females	Males	Females	Males	Females
Breakfast	1.90±0.64	1.46±0.61*	49±17 (44%)	39±15 (46%)*	20±8 (18%)	14±7 (16%)*	18±8 (36%)	14±7 (36%)*
Lunch	2.17±0.80	1.59±0.62*	56±22 (44%)	42±17 (45%)*	23±11 (18%)	16±7 (17%)*	21±9 (37%)	15±8 (36%)*
Dinner	1.75±0.45	1.51±0.45*	53±16 (51%)	46±13 (51%)*	21±6 (20%)	19±6 (21%)	12±5 (26%)	10±5 (26%)
Snacks	0.80±1.06	0.54±0.30	30±30 (63%)	24±14 (73%)	3±5 (6%)	2±2 (6%)	6±14 (30%)	3±3 (20%)

Values are mean±SD. Data were analyzed by a Students t-test. * Indicates a significant difference between males and females, P<0.05

### Protein provision and consumption

Protein provision and consumption (g•kg^-1^•d^-1^) from selfselected hospital meals during hospitalization are presented in Figure 2. Self-selected hospital meals provided 0.75 ± 0.16 g•kg^-1^•d^-1^ in males (Figure 2A) and 0.79±0.21 g•kg^-1^•d^-1^ in females (Figure 2B) during hospitalization, while actual protein consumption averaged merely 0.59±0.18 and 0.50±0.21 g•kg^-1^•d^-1^, respectively. The consumed amount of protein was 32±19% lower than the provided amount of protein at all days of hospitalization (P<0.001). Protein intake levels increased during hospitalization to a similar extent in males and females (time x gender interaction effect, P=0.306, main time effect, P<0.001), with lower protein intakes on the day of surgery (day 1) when compared with day 2 and day 3 (P<0.001), and on day 2 when compared with day 3 (P=0.007). Protein intake during hospitalization was overall lower in females when compared with males (main gender effect, P=0.028).

### Protein intake per meal

Distribution of protein provision and consumption (g) across main meals is presented in Figure 3. Absolute protein intake across main meals is shown in Table 2. Self-selected hospital meals provided 26±8, 28±10, and 23±4 g protein in males, and 23±7, 23±6, and 24±4 g protein in females at breakfast, lunch, and dinner, respectively. Protein consumption was 32±23% lower than protein provision at breakfast (P<0.001), 27±23% at lunch (P<0.001), and 17±18% at dinner (P<0.001). A significant time x gender interaction effect was observed for protein intake distribution during hospitalization (P<0.001). Protein intake in males averaged 20±7, 22±11, and 21±6 g at breakfast, lunch, and dinner, respectively, with no differences between main meals (P=0.157). Protein intake in females differed between main meals and averaged 14±7, 16±7, and 19±6 g, respectively (P<0.001). Post-hoc analyses in females showed that protein consumption was lower at breakfast and lunch when compared with dinner (both P<0.001), and lower at breakfast when compared with lunch (P=0.042). Protein consumption during hospital admission was lower in females when compared with males at breakfast and lunch (both P<0.001), but did not differ at dinner between genders (P=0.120). Snack consumption provided merely 3±5 g protein per day in males and 2±2 g in females, with no differences between genders (Figure 3A and B; P=0.141). Protein distribution per main meal as % of the total consumed amount of protein is presented in Supplemental Figure 1. Breakfast, lunch, and dinner provided 30±7, 33±8, and 33±7% protein in males (Supplemental Figure 1A) and 27±8, 30±7, 39±9% protein in females (Supplemental Figure 1B). Snacks contributed for the remaining 4±5% protein in males and 4±3% in females.

### Protein sources

**Figure 4:**
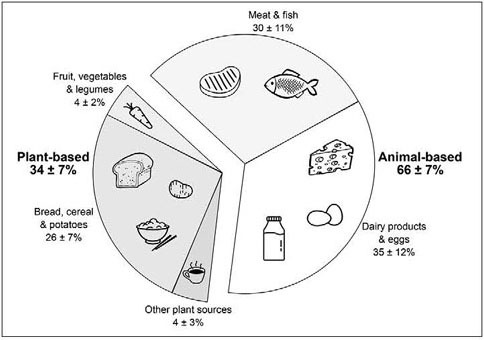
Contribution of animal- and plant-based sources to total dietary protein intake (expressed as a percentage of total protein intake) in older, hospitalized patients (n=101)

The contribution of animal- and plant-based sources to total protein intake (%) is shown in Figure 4. In total, protein intake from self-selected hospital meals contained a higher amount animal-based protein sources when compared with plant-based protein sources (66±7% and 34±7%, respectively, P<0.001). Dairy products and eggs provided the largest amount of animal-based proteins (35±12%), followed by meat and fish (30±11%). For plant-based protein sources, bread, cereals, and potatoes provided the largest amount of protein (26±7%). Fruit, vegetables, and legumes provided only 4±2% of plant-based protein to the total diet, and 4±3% did not fit in any of these categories.

## Discussion

Self-selected hospital meals provided patients with ~0.8 g•kg^-1^•d^-1^ protein during short-term hospitalization, while actual protein intake was below 0.6 g•kg^-1^•d^-1^ with more than 30% of the provided food being discarded. Absolute protein intake per main meal ranged from 14 to 22 g in all patients, with protein intake being particularly low at breakfast in females. A total of 34% of daily protein intake was derived from plant-based protein sources.

In the present study, we assessed actual energy and protein consumption patterns during short-term hospitalization in older patients admitted for elective hip and knee arthroplasty. Daily energy consumption averaged 5.29±1.77 and 3.96±1.39 MJ•d^-1^ in males and females, respectively, which is merely ~50% of the estimated energy requirements. Consequently, all patients remained in a negative energy balance throughout their hospitalization. At present, up to 30% of the older patients in Western-European hospitals have been identified as being malnourished during hospitalization (9-11, 44, 45). Poor nutritional status in older hospitalized patients has been associated with accelerated weight loss, slower wound healing, an increase in length of hospital stay, higher mortality rates, and more frequent hospital readmission (9-11, 45, 46). An energy deficit during hospitalization and bedrest accelerates the loss of skeletal muscle mass (21). The muscle atrophy typically observed during hospitalization (19, 20, 47) may be largely attributed to the lack of sufficient protein consumed as a result of low(er) energy intake levels. We assessed dietary protein consumption in older patients during short-term hospitalization after total hip or knee arthroplasty. Self-selected hospital meals provided patients with merely ~0.8 g•kg^-1^•d^-1^ protein, which is well below recommended daily intake levels of 1.2 g•kg^-1^•d^-1^ (25, 26). Since 30% of food provided was discarded, actual protein consumption was much lower and averaged 0.6 g•kg^-1^•d^-1^. Protein intake was particularly low on the day of surgery when compared with subsequent hospitalization days (Figure 2). Though protein consumption increased on the following days of recovery on the orthopedic ward, protein consumption still remained far below recommended daily intake levels (1.2 g•kg^-1^•d^-1^) throughout the entire hospitalization period. This seems to support previous work showing that protein consumption is insufficient in older patients during shortterm hospitalization (36, 39). Consequently, interventional strategies should be employed to effectively increase energy intake to match energy requirements and, as such, avoid energy malnutrition. Furthermore, protein intake should be maintained at habitual intake levels, which in the light of a reduced daily energy intake, requires the installment of a more protein-dense diet.

The ingestion of dietary protein, and more specifically the postprandial rise in plasma amino acid concentrations, stimulates muscle protein synthesis and inhibits muscle protein breakdown, thereby stimulating muscle protein accretion (48). The postprandial increase in muscle protein synthesis rates forms an important factor in muscle mass maintenance. Ingestion of 20 g of a high quality protein has been shown to increase postprandial muscle protein synthesis rates in young adults (49, 50). Due to anabolic resistance with aging, greater amounts of protein (>20 g per meal) are required to significantly stimulate muscle protein synthesis in older individuals (51-53). In the present study, the amount of protein consumed at breakfast, lunch, and dinner varied between 14 and 22 g in both the male and female patients (Figure 3). While protein intake per meal was likely sufficient to induce an anabolic response in the male patients, protein intake remained well below 20 g for most meals in females. Particularly at breakfast, protein intake averaged only 14±7 g in the female patients. This seems to agree with previous findings showing that protein consumption is particularly low at breakfast in older individuals (22, 24, 54). Given the suboptimal anabolic response with every main meal and the presence of anabolic resistance to protein ingestion in older individuals (53), the protein content of each main meal should be increased to allow ingestion of at least 20 g protein per main meal.

As animal-based proteins are generally regarded as being more anabolic than plant-based proteins (55, 56), we also assessed the contribution of animal- and plant-based protein sources to total dietary protein intake (Figure 4). Self-selected hospital meals contained a relative large amount of animalbased protein sources (66±1%) when compared with plantbased protein sources (34±1%). This is in line with the general Western diet in community-dwelling older adults (57). As shown in Figure 4, protein intake in older patients was mainly derived from meat, fish, dairy products and eggs, which are the main protein sources in the aging population (6, 58, 59). The relative contribution of animal versus plant-based protein sources in the hospital diet does not seem to require any modification.

Our data clearly show that even healthy patients admitted for elective hip or knee arthroplasty consume far less energy and protein than the estimated daily requirements. Since more than 30% of the provided food is not consumed, it is obvious that simply increasing food provision will not be effective to prevent energy and protein malnutrition during hospitalization. As the maintenance of habitual protein intake levels is key to attenuate muscle mass loss, a more protein-dense diet should be consumed as total energy intake is typically reduced during hospitalization. Various strategies can be applied to increase the relative protein intake in the diet, including the consumption of more protein-rich foods, supplementation with oral nutritional supplements (ONS), fortification of meals with protein isolates, and/or the provision of well-timed protein-rich snacks (36, 38, 60, 61). To increase absolute protein intake in the diet, a first target for intervention should be breakfast. As protein consumption is typically low at breakfast (Figure 3), increasing protein intake at breakfast should be of greater benefit. In support, additional protein supplementation at breakfast has been shown to increase skeletal muscle mass and function in older, frail individuals (62, 63). Another target would be increasing food consumption on the day of surgery. As energy and protein intake were hardly existing on the day of surgery, food provision during the pre- and post-surgery period could be installed within the restraints set by the surgical procedures. The provision of ONS may help to cover the energy and protein deficits during the first 1–2 days following surgery. During subsequent days of hospitalization, food fortification, provision of more protein-dense foods, and/or an adding an extra protein meal will likely be more appropriate. In line, provision of protein-enriched foods (such as bread, yoghurt, cake, fruit juice, and soup) or the use of more protein-dense foods throughout the day have been shown effective in improving protein intake during hospitalization (36, 38, 60). In addition, the timing of protein-rich products serves as alternative strategy to increase protein intake levels during hospitalization. We have recently shown that protein ingestion prior to sleep increases overnight muscle protein synthesis rates in healthy, older men (64, 65), and supports muscle mass and strength gains during prolonged exercise training in young adults (66). However, whether prolonged pre-sleep protein supplementation can attenuate muscle mass and/or strength loss in older patients during hospitalization remains to be assessed. Nutritional intervention strategies need to be assessed for their efficacy to increase energy and protein intake and, as such, to help preserve muscle mass and strength in older patients during hospitalization.

In conclusion, energy and protein intake levels remain well below requirements during hospitalization in older patients admitted for elective hip or knee arthroplasty. While patients are provided with 0.8 g protein g•kg^-1^•d^-1^, actual protein consumption does not even reach 0.6 g•kg^-1^•d^-1^ with 30% of the provided food being discarded. Strategic interventions are required to increase energy intake and ensure maintenance of habitual protein intake levels in older patients admitted for elective orthopedic surgery.

*Acknowledgements:* We thank all medical staff and meal assistants of the Orthopedic nursing ward of the Maastricht University Medical Centre+ for their collaboration and assistance.

*Author contributions:* MEGW, IWKK, AAJV, PG, and LJCvL designed the study. MEGW and IWKK conducted the data collection with the assistance and expertise of PG, RM, JAG, and PJE. PG and AAJV provided the nutritional specifications of the products and suppliers. MEGW and IWKK performed the (statistical) analysis of the data together with LBV and LJCvL. MEGW, IWKK, and LJCvL wrote the manuscript and had primary responsibility for final content. All authors read and approved the final manuscript.

*Ethical standards:* Observational food intake data and retrospective, blinded patient data were collected under the Agreement on Medical Treatment Act and the Personal Data Protection Act, according to Medical Ethical standards.

*Clinical Trial Registration Number:* NTR5942 (www.trialregister.nl)

*Conflict of Interest:* AAJV is an employee of Daily Fresh Food B.V., Geleen, the Netherlands. Daily Fresh Food B.V. is a manufacturer and supplier of fresh food products and convenient meal components for healthcare and consumer services. PE has received research grants of Zimmer-Biomet, Regentis, Active Implants, DSM Biomedical, DePuySynthes, and is a shareholder of Chondropeptix B.V. IWKK and LBV have received speaking honoraria from Nutricia Research. LJCvL has received research grants, consulting fees, and/or speaking honoraria from Friesland Campina and Nutricia Research. None of the other authors had any personal or financial conflict of interest.

*Funding:* This research did not receive any funding from agencies in the public, commercial, or not-for-profit sectors.

## Electronic supplementary material

Supplementary material, approximately 109 KB.
