# Supplementary figures and images for: Protein Intake Falls below 0.6 g·kg-1·d-1 in Healthy, Older Patients Admitted for Elective Hip or Knee Arthroplasty
Source: J Nutr Health Aging. 2019 Jan 23;23(3):299–305. doi: 10.1007/s12603-019-1157-2 (PMC6399806; doi:10.1007/s12603-019-1157-2)

A

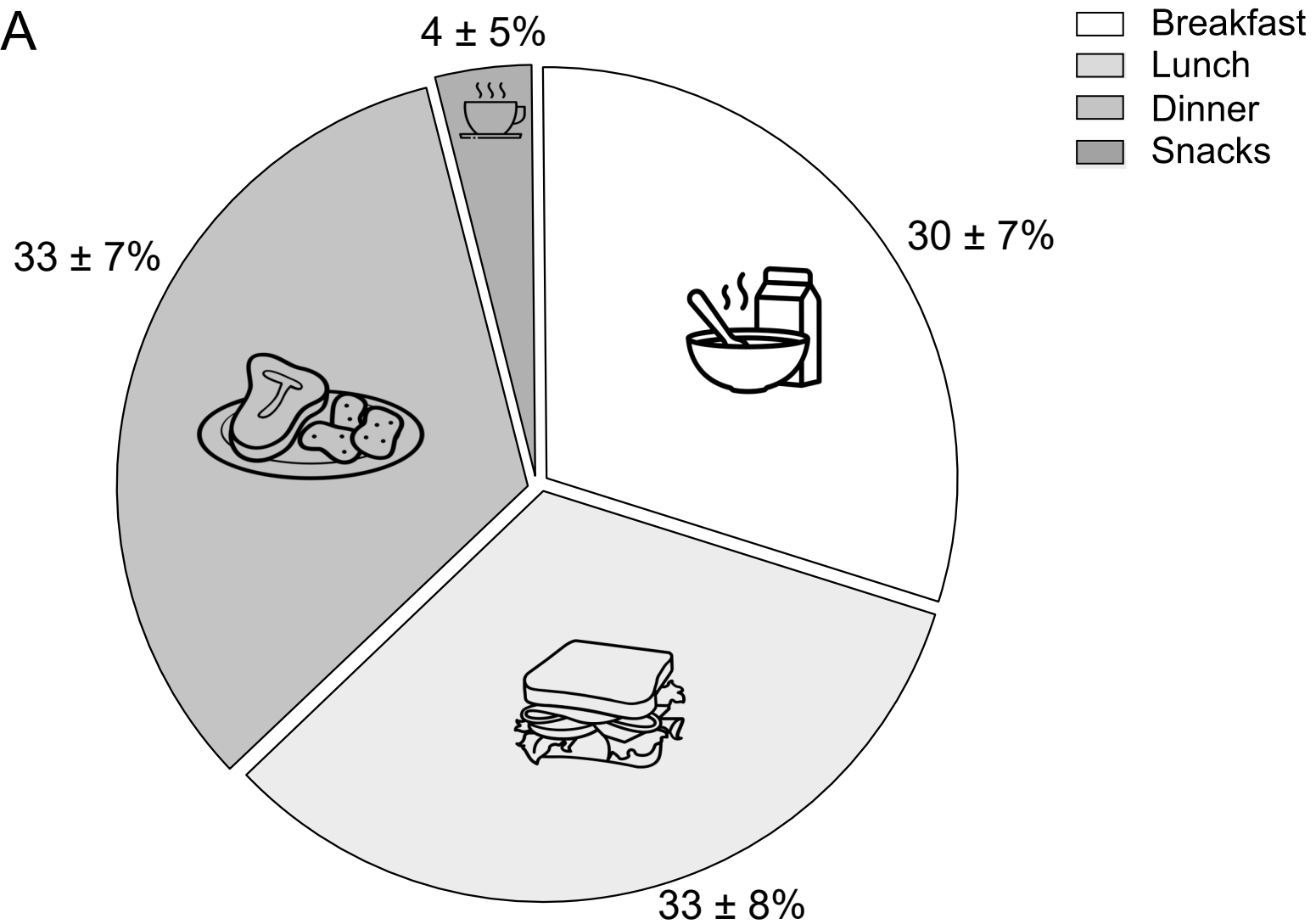

B

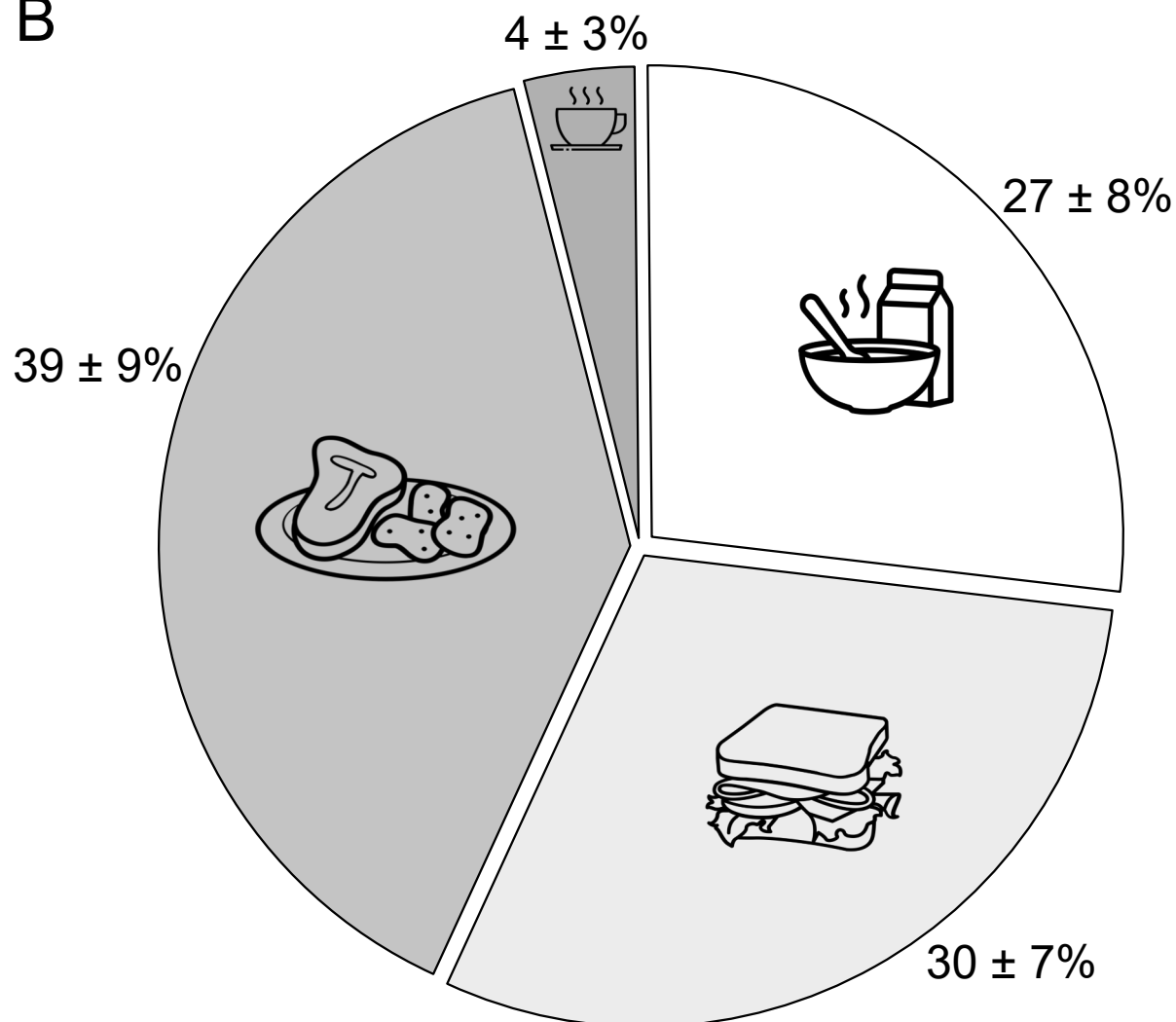

Supplement: Supplementary file 1 — Supplementary material, approximately 109 KB. [file mmc1.pdf]
