# Supplementary material for: Protein Intake Falls below 0.6 g·kg-1·d-1 in Healthy, Older Patients Admitted for Elective Hip or Knee Arthroplasty
Source: J Nutr Health Aging. 2019 Jan 23;23(3):299–305. doi: 10.1007/s12603-019-1157-2 (PMC6399806; doi:10.1007/s12603-019-1157-2)
Supplement: Supplementary file 1 [file 12603_2019_1157_MOESM1_ESM.pdf]

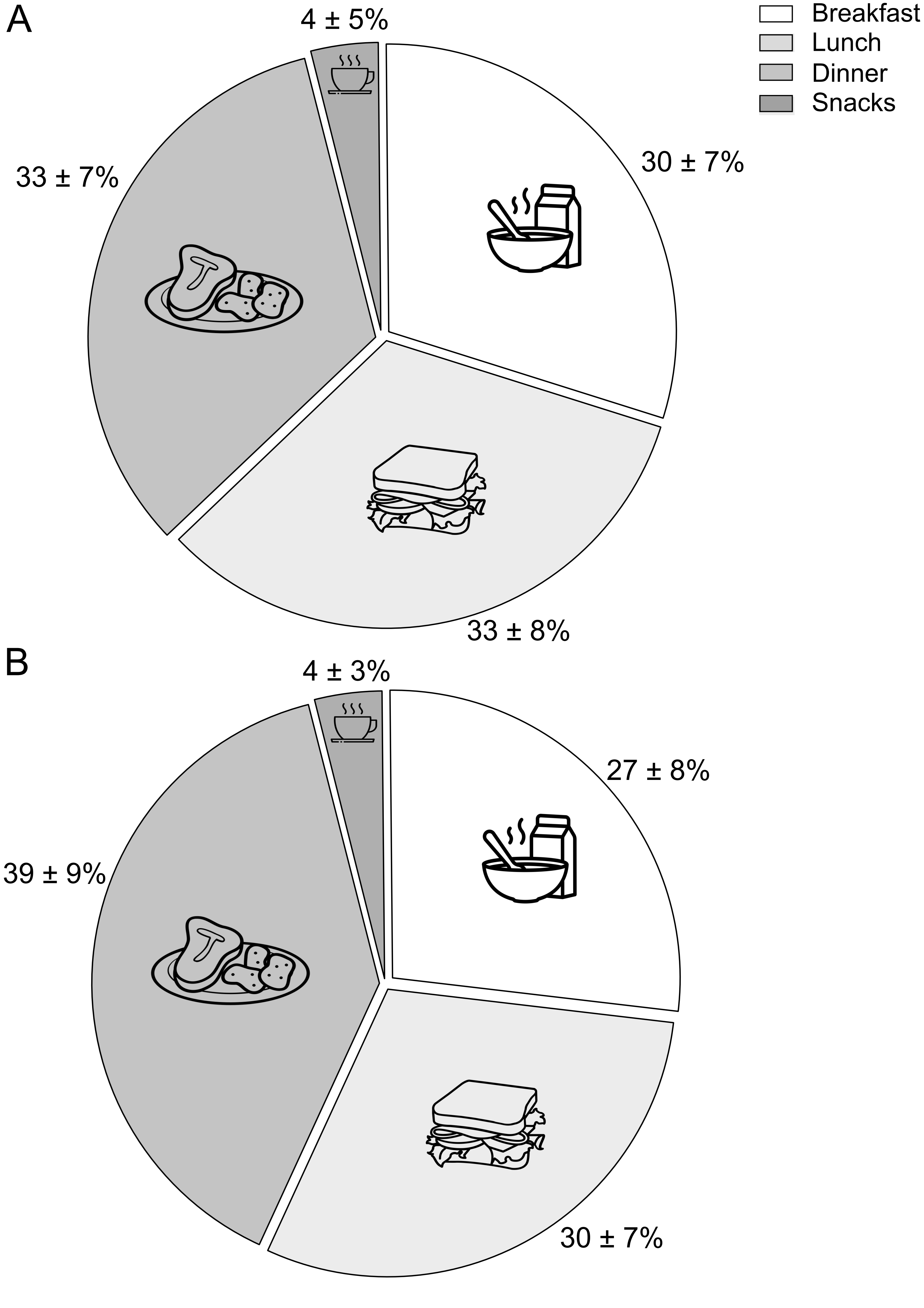

A
4 ± 5%
30 ± 7%
33 ± 7%
33 ± 8%
B
4 ± 3%
27 ± 8%
39 ± 9%
30 ± 7%
Breakfast
Lunch
Dinner
Snacks
